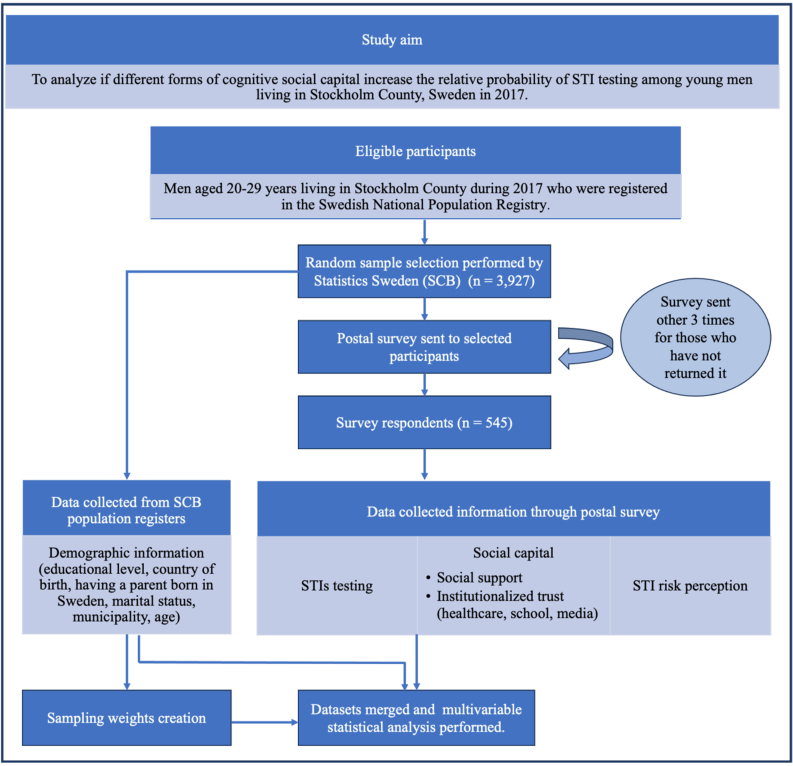# Cognitive social capital as a health-enabling factor for STI testing among young men in Stockholm, Sweden: A cross-sectional population-based study

**DOI:** 10.1016/j.heliyon.2023.e20812

**Published:** 2023-10-08

**Authors:** Ana Paula Finatto Canabarro, Malin Eriksson, Anna Nielsen, Zangin Zeebari, Mariano Salazar

**Affiliations:** a Department of Global Public Health, Karolinska Institutet, Tomtebodavägen 18a, Widerströmska Huset, 171 77, Stockholm, Sweden; b Department of Social Work, Umeå University, 901 87, Umeå, Sweden; c Jönköping International Business School, Jönköping University, Gjuterigatan 5, 553 18, Jönköping, Sweden

**Keywords:** Social capital, HIV testing, Sexually transmitted diseases, Sweden, Social support, Trust

## Abstract

**Objective:**

To assess whether different forms of cognitive social capital increased the relative probability of testing for sexually transmitted infections (STIs) among young men living in Stockholm, Sweden.

**Methods:**

A population-based cross-sectional study was conducted in 2017 with men aged 20–29 years living in Stockholm County, Sweden (n = 523). The main outcome was STI testing patterns (*never tested, tested only within a12-monthperiod, tested only beyond a12-monthperiod, repeatedly tested*). The main exposure were two forms of cognitive social capital: social support (*having received help*, *having someone to share inner feelings with*) and institutionalized trust (in *school, healthcare, media*). Data were analyzed using weighted multivariable multinomial logistic regression to obtain adjusted weighted relative probability ratio (aRPR).

**Results:**

After adjusting for confounding factors, receiving help (aRPR: 5.2, 95% CI: 1.7–16.2) and having someone to share inner feelings with (aRPR: 3.1, 95% CI: 1.2–7.7) increased the relative probabilities of young men testing for STIs, but only for those testing beyond a 12-month period. Trust in media increased the relative probability of STI testing for those testing only within a 12-month period (aRPR: 2.6, 95% CI: 1.1–6.1) and for those testing repeatedly (aRPR: 3.6, 95% CI: 1.5–8.8).

**Conclusion:**

Young men in Stockholm County exhibit distinct STI testing patterns. Social support and trust in media were factors that increased the probability of being tested for STIs, with this effect varying according to the young men's STI testing pattern. Further studies are required to explore how trust in media might promote STI testing in this population.

## 1 Introduction

Sexually transmitted infections (STIs) represent one of the most significant global public health concerns [1]. In 2016 alone, 376 million new cases of curable STIs (gonorrhea, syphilis, chlamydia, and trichomoniasis) were estimated worldwide [2],pp63]. Human immunodeficiency virus (HIV) and other STIs have been identified as the leading causes of death among individuals aged 15–49 years worldwide among communicable diseases since 1997 [3].

Globally, STIs (including HIV) result in 15.03 deaths per 100,000 population and 887.33 per 100,000 disability-adjusted life years [3]. Due to its worldwide impact and negative health consequences, ending HIV epidemic by 2030 is a key target included in the United Nation's Sustainable Developmental Goals (2015–2030) [4] and Joint United Nations Programme on HIV/AIDS Global AIDS Strategy (2021–2026) [5],pp24].

In Sweden, the most common STI is chlamydia. Between 2015 and 2016, approximately 36,000 new cases of chlamydia were reported, accounting for 94% of new STI cases in the country [6]. Approximately 55% of all chlamydia cases registered in 2019 in Sweden occurred among young people aged 20–29 years, and Stockholm County accounted for nearly one-third (9,930) of the total cases in the same year [7]. Concerningly, despite a higher incidence of chlamydia infection in males compared to females (7.1% vs. 4.2% in 2017), only 31% of those who were tested during this period were males [8].

Untreated STIs can lead to adverse health outcomes, even when asymptomatic, resulting in long-term health consequences for both men and women, as well as their children. These complications include pelvic inflammatory disease, ectopic pregnancies, epididymitis, infertility, neonatal and infant infections, and even increased risk of central nervous system diseases and cancer, among others [9]. To mitigate these negative consequences, health systems must implement preventive measures, as well as early detection and treatment policies.

Testing for STIs has been associated with various individual and health system factors. At the individual level, factors that facilitate testing include older age, educational status, knowledge of, and perceived susceptibility to STIs [10], number of sexual partners, and history of previous STI [11], among others. Being male has been found to be negatively associated with STI testing [8,12]. At the health system level, factors that promote STI testing include the accessibility, confidentiality, and flexibility of health services, especially for young people [13].

In Sweden, the STIs surveillance system aims to minimize the spread of STIs and their adverse consequences at individual and populational levels [14],pp16]. It consists of opportunistic testing of both symptomatic and asymptomatic individuals and contact tracing [15]. STI testing and treatment are free for young people and subsidized for other age groups [16]. There is no clear guideline on STI testing frequency among asymptomatic individuals in Stockholm County; however, the recommendation for healthcare providers is to focus their resources on sexual risk groups [6].

The Swedish STI surveillance system has proven to be successful in tracing infected sexual partners and notifying positive cases compared to other European countries [17]. However, the system still faces significant challenges, such as gender inequalities in service utilization, with men being tested for STIs less frequently than women. One reason for this disparity might be the design of the healthcare system. In Sweden, women have more opportunities for STI testing compared to men since testing is offered when women access contraceptive services, whereas men lack a parallel service where they can be reached. Furthermore, even when men are notified that a sexual partner has tested positive for an STI and are requested to undergo testing – a protocol adopted in Sweden for STI contact tracing – they are less likely than women to visit the sexual health clinics and get tested [18].

### 1.1 Social capital, health and STI testing

Social capital is a key health determinant [19],pp295]. It concerns “*social networks, the reciprocities that arise from them and the value of these for achieving (mutual) goals*” [20],pp307]. In other words, it represents the resources embedded within social networks. Social capital can influence health by providing access to various individual and group resources. There is not a single definition of social capital [21]. Social capital can be understood as either an individual [22] or a group attribute [23,24].

In this paper, we adopt the perspective of social capital as an individual attribute. Following this perspective, Bourdieu argues that individuals can gain access to various resources through their membership in social networks [22]. However, inclusion in these networks often occurs through the exchange of values, hence individuals with fewer resources are less easily invited into valuable social networks. Thus, social capital is not equally accessible to everyone but rather a result of one's social position and status in society [22].

To ease the conceptualization and study of social capital, it has often been divided into two primary domains: structural and cognitive social capital. *Structural* social capital refers to actual involvement in social networks (i.e., social participation) and can be measured in terms of the frequency or degree of participation [25]. In this paper, we focused on measuring *cognitive* social capital, which refers to the intangible aspects of social network involvement, such as perceptions about trust, reciprocity, and various forms of support (e.g., emotional and instrumental) [25]. Trust can be further divided into trust in people in general (generalized), trust in people one knows (personalized), and trust in institutions (institutionalized) [26].

Social capital can influence STI testing through different pathways, including social influence, access to social support, and/or social participation [27]. For instance, young men's attitudes towards STI testing can be influenced by their peers' attitudes (social influence pathway). Such influence can lead to health-enhancing or health-damaging behaviors, depending on the norms embraced [27]. Additionally, social support from young men's peers could help reduce stress related to STI testing (social support pathway). Social capital can also serve as a channel for accessing key health information (social participation pathway).

The association between health and social capital varies depending on the type of social capital, the health outcome measured, and population group under study [28], making the impact of social capital on health difficult to predict. High levels of social capital have been associated with improved physical and mental health [29], healthier lifestyles [30], and increased preventive health seeking behaviors [31]. However, social capital can also exert a negative influence on health when group norms endorse negative health behaviors, as seen in some traditional forms of masculinity (e.g., “no one here attends health check-ups because that is a sign of weakness”) [32].

Social capital has also been associated with men's sexual and reproductive health. Studies have found that high social capital levels decreased men's risk for acquiring STIs [33] and facilitated HIV testing among men who have sex with men (MSM) [34]. However, contradictory results have also been reported. In a systematic review, McPherson et al. reported that peer role models can either increase or decrease adolescents sexual risk-taking depending on the group's characteristics [30].

Previous studies on STIs in Sweden [7,35] and elsewhere [36,37] have provided valuable insights into STI testing prevalence and its risk factors. However, none of these studies have assessed whether different forms of cognitive social capital can increase testing rates among young men living in Stockholm County. Our study will contribute to fill this knowledge gap and provide valuable insights that can be used to enhance intervention programs aimed at increasing STI testing in this population.

## 2 Objectives

The study aims to analyze whether different forms of cognitive social capital increase the relative probability of STI testing among young men living in Stockholm County, Sweden in 2017.

## 3 Methods

A population-based cross-sectional postal survey was conducted with men aged 20–29 years living in Stockholm County in 2017. Stockholm County comprises 26 municipalities and represents approximately 23% of the Swedish population [38].

This study used data that had previously been collected for the CTEST project (*CTEST: Improving young Swedish men health service utilization for Chlamydia Trachomatis infection, detection and treatment. How can we do it?*). The CTEST project aimed to identify individual, social and health service factors facilitating/hindering young men's health service utilization for *Chlamydia trachomatis* detection in Sweden (personal communication with M. Salazar on February 1st, 2021).

The sample size was calculated using a standard formula for cross-sectional studies [39]:

$$n=\left[\text{DEFF}∙\text{Np}\left(1-p\right)\right]/\;\left[(d^{2}/Z_{1-\alpha /2}^{2}∙\left(N-1\right)+p∙\left(1-p\right)\right]$$

The following parameters were used: population size (N) of one million; hypothesized frequency of the outcome in the population (p) of 20% [40]; confidence limit of 2%; and design effect (DEFF) of 1. The frequency of the outcome, which consisted of the lifetime STI testing, used data from a Norwegian study reporting lifetime STI testing prevalence for young men aged 20–25 years [40]. This was considered appropriate since both Sweden and Norway share similar sociodemographic characteristics. The preliminary sample was 1535 individuals. Further adjustment for a non-response rate of 60% (based on the expected low response rate typical of this population [41]) yielded a final sample size of 3927 young men.

The sampling and data collection were conducted in collaboration with Statistics Sweden (SCB) [42]. The final sample was selected using a random sampling procedure from men aged 20–29 years living in Stockholm County during 2017 who were registered in the Swedish National Population Registry. Outcome and main exposures variables were collected through a postal survey and the corresponding young men's demographic characteristics were obtained from the Swedish National Population Registry. The postal survey was collected between September 25th and October 30th, 2017. For those who did not respond to the first wave of questionnaires, two postal reminders were sent, one every two weeks (Oct.9th and 23rd respectively). The last reminder also included an additional copy of the questionnaire.

The original survey was written in Swedish and was translated into English solely for this report. Out of the 3927 selected individuals, 545 answered the survey. Twenty-two respondents reported never having had sex and were consequently excluded from the analyses. Once the survey data on the main outcome and exposure variables were collected, it was merged with corresponding sociodemographic data from the Swedish National Population Registry and anonymized for analysis.

### 3.1 Variables

#### 3.1.1 STI testing patterns

The outcome variable was STI testing patterns. It was constructed using two questions from the survey: “*Have you tested yourself, or bought a test, for a sexually transmitted infection (ex.: chlamydia, gonorrhea, syphilis, HIV, trichomonas or other) in some of the following places?”* (listed places: “*Youth Health Clinic*”, “*Primary healthcare center*”, *Private healthcare clinic*”, “*Hospital/Infectious disease clinic/Venereal disease clinic*”, “*Non-cost chlamydia test available online*”, “*Chlamydia test available at the pharmacy*”, “*Other*”) and “*Have you tested, or bought a test, specifically for chlamydia?”*. The answer options for both questions were “*Never*”; “*Yes, during the last* *12 months*”, and “*Yes, over* *12 months* *ago*”. Participants could choose more than one response (e.g., if they had been tested both during the last months and over 12 months ago or in different places, they could check both options). The questions are presented in Appendix 1 as supplementary material.

We combined the above data and created four STI testing patterns: “*Never tested*”, “*Tested only within a* *12-month* *period*”, “*Tested only beyond a* *12-month* *period*”, and “*Repeated tester (Tested both beyond and within a* *12-month* *period)*”. The authors aimed to study single testers separately from repeated testers. Previous studies in the same study population and setting have suggested that repeated testers may have different sexual behaviors and motivations for being tested compared to single testers [43,44]. Dichotomizing the outcome into never and ever tested could potentially mask these differences. Therefore, it was important to analyze the data separately for single testers and repeated testers to better understand the factors that drive STI testing in these two groups.

#### 3.1.2 Social capital

Cognitive social capital (social support and institutional trust) was measured using questions from a scale developed by Eriksson et al. [26], which had previously been used in population-based studies in Sweden (both sexes; 18–84 years old). The original scale was shortened to include only items relevant to the research question or items that had been shown to be associated with STI testing in previous studies, such as perceptions of instrumental and emotional social support [34] and trust in institutions (healthcare, school, and media) [45].

The following questions were used to measure perceptions of instrumental and emotional support and were used as exposure variables: *“In the last* *12 months, has a friend or acquaintance helped or performed any service for you?*” (*Yes/No*) and *“Do you have someone you can trust and share your innermost feelings with?”* (*Yes/No*). Trust in institutions was assessed using the question *“What level of confidence do you have in the following institutions in society?”* (*Healthcare, School,* and *Media*) (see Supplementary material, Appendix 1). Each institution was analyzed individually. The response options were *“Very little”, “Quite little”, “Quite a lot”,* and *“A lot”* and were dichotomized into *“Low”* (combining *“Very little”* and *“Quite little”* options) and *“High”* (combining *“Quite a lot”* and *“A lot”* options) for analysis.

#### 3.1.3 Demographic data

The variables age (years); born in Sweden (*Yes/No*); either parent born in Sweden (*Yes/No*); educational level (*Up to 9 years/Between 10 and 12 years/University level <3 years/University level ≥ 3 years*); marital status (*Married/Not married*), and self-perceived risk of contracting STIs (*No risk/Low* *risk/Medium* *risk*/*High risk*) were treated as confounders and included in all multivariable models.

### 3.2 Data analysis

Missing data ranged from 0.19% to 4.78% in the outcome questions, 0%–1.53% in the exposure questions, and 0%–8.8% in the confounder variables. The confounder “*Either parent born in Sweden*” had the highest missingness. We conducted a sensitivity analysis and found that imputing the missing data in the multivariable analysis did not modify the estimates. Thus, the multivariable analysis includes only complete cases.

Probability weights were included in the analysis to mitigate the risk of selection bias due to low response rate and ensure that the survey results accurately represented the population ([46], pp343-344). Probability weights were calculated based on the probability of responding to the postal survey, considering the overall sample's age, country of birth, county, educational level, marital status, and whether either parent was born in Sweden ([46], pp343-344).

To create the probability weights, we initially conducted a logistic regression with a dichotomous variable as the outcome, indicating whether the individual responded to the postal survey (options: *Yes/No*), and using all the aforementioned demographic variables as exposures. The regression results were saved as the predicted probability of responding to the survey. Next, we created the weights as the reciprocal of the predicted probabilities of responding to the survey. This method ensured that the weights considered the proportion of individuals in the sample with specific characteristics, such as educational level, compared to the total number of individuals with those characteristics among the survey respondents. Probability weights were included in all analyses.

Chi-squared and Student's t-tests assessed the bivariate differences between unweighted groups, while the F-test was applied to weighted values. Chi-squared and F-tests also estimated the difference in each demographic characteristic and exposure among the STI testing patterns strata.

Weighted multivariable multinomial logistic regressions were used to estimate the potential relationship between different forms of social capital and STI testing patterns. Results are presented as crude weighted relative probability ratios (RPR) and adjusted weighted relative probability ratios (aRPR) with 95% confidence intervals (CI). Variables that have been shown in previous studies to be associated with both social capital [47] and STI testing [12] were included in the analysis as confounders. The Margins command (STATA/IC 16.1 – StataCorp, College Station, TX) was used to compute predicted probabilities and 95% CI of social capital variables that were found to be significant in the multivariable analysis.

The population attributable fraction (PAF) was calculated to estimate the contribution of social capital aspects to the relative probability of being tested for STIs in each testing patterns. PAF was calculated only for the statistically significant estimates and was guided by the formula:

PAF=p∙(1−1/PR)

where *p* (proportion) consisted of the ratio of exposed cases among all cases (the proportion of people with high social capital in each testing patterns), and PR (probability ratio) of the aRPR obtained from the regressions [48]. Results are presented in percentages.

The models were assessed for multicollinearity using tolerance and variation inflation factor (VIF) post estimation tests. Multicollinearity was defined as tolerance values below 0.1 and VIF values equal to or greater than 10. The results did not indicate multicollinearity in the weighted multivariable multinomial logistic regression model (VIF: 1.36) nor among the cognitive social capital variables (highest VIF: 1.87; lowest tolerance: 0.53).

Analyses were performed using STATA/IC 16.1 (StataCorp, College Station, TX) and considered statistically significant if p < 0.05. See Appendix 2 in the supplementary material section for a description of the methodological steps of this study.

### 3.3 Ethical considerations

Ethical approval was obtained from Stockholm Regional Ethics Committee (reference number 2017/833-31/5). Participation was voluntary, and the survey included the essential elements of informed consent. All datasets were collected and previously anonymized by Statistics Sweden. Therefore, there was no information that allowed the researchers to identify participants or link sensitive information to the participants.

## 4 Results

### 4.1 Descriptive data

The description of respondents and non-respondents is presented in Table 1. All demographic characteristics except marital status were statistically different between respondents and non-respondents (p < 0.05). Respondents reported a higher educational level (University level ≥3 years: 25.4%, 131/516), were mostly born in Sweden (84.9%, 462/544), had at least one parent born in the country (84.5%, 421/498), were living in Stockholm municipality (48.6%, 265/545), and were older (mean age: 25.3 years) compared to non-respondents (Table 1, p < 0.05). Participants reported an overall high trust in healthcare (76.7%) and school (64.8%), and low trust in media (30.2%) (data not shown).

**Table 1:** Description of the respondents' and non-respondents' demographic profiles and unweighted prevalence of respondent answers to the survey, Stockholm County, Sweden, 2017 (N = 3927^a^).

	Respondents	Non-respondents	Total
	n (%)	n (%)	N (%)
All	545 (13.9)	3382 (86.1)	3927 (100)
Educational Level, n = 3495^b^			
Up to 9 years	78 (15.1)	620 (20.8)	698 (20.0)
Between 10 and 12 years	179 (34.7)	1553 (52.1)	1732 (49.6)
University level <3 years	128 (24.8)	487 (16.3)	615 (17.6)
University level ≥3 years	131 (25.4)	319 (10.7)	450 (12.9)
Country of birth [Sweden], n = 3921^b^	462 (84.9)	2530 (74.9)	2992 (76.3)
Either parent born in Sweden [Yes], n = 3384^b^	421 (84.5)	2086 (72.3)	2507 (74.1)
Marital status [Married], n = 3923	29 (5.3)	208 (6.2)	237 (6.0)
Municipality [Stockholm], n = 3927^b^	265 (48.6)	1461 (43.2)	1726 (43.9)
Age (years), mean (SD), n = 3923^c^	25.3 (2.8)	25.0 (2.8)	25.0 (2.8)
Self-perceived STI risk, n = 521			
No risk	315 (60.5)	NA	NA
Low risk	137 (26.3)	NA	NA
Medium or high risk	69 (13.2)	NA	NA
Social support			
Having received help from someone [Yes], n = 523	489 (93.5)	NA	NA
Having someone to share inner feelings with [Yes], n = 516	446 (86.4)	NA	NA
Trust in institutions		NA	NA
Trust in healthcare [High], n = 518	418 (80.7)	NA	NA
Trust in school [High], n = 517	355 (68.7)	NA	NA
Trust in media [High], n = 509	239 (46.9)	NA	NA
STI testing patterns, n = 523			
Never tested	228 (43.6)	NA	NA
Tested only within a 12-month period	194 (37.1)	NA	NA
Tested only beyond a 12-month period	61 (11.7)	NA	NA
Repeated testers^d^	40 (7.6)	NA	NA

a The total number of participants in each variable might vary due to missing data.

b Statistically significant (p < 0.05), Chi-squared test.

c Statistically significant (p < 0.05), Mann-Whitney *U* test.

d Tested both beyond and within a 12-month period.

### 4.2 Bivariate analyses

The most common testing patterns were those who have never tested (45.2%) followed by those testing only beyond a 12-month period (33.8%) (Table 2). The bivariate weighted analyses presented in Table 2 showed that educational level varied between testing patterns with those testing beyond a 12-month period having higher educational levels than the other STI testing categories. Those testing beyond a 12-month period were also older than the other STI testing categories. The percentage of people living in Stockholm municipality was higher among those testing both beyond and within a 12-month period than among the other STI testing categories. The percentage of young men who had either parent born in Sweden was lower among those testing only within a 12-month period than among the other STI testing categories (Table 2, p < 0.05).

**Table 2:** Weighted characteristics of survey respondents stratified by sexually transmitted infections (STI) testing patterns presented in percentage (%) and 95% confidence intervals (CI); Stockholm County, Sweden, 2017 (n = 468).

	STI testing pattern			
	Never tested	Tested only beyond a 12-month period	Tested only within a 12-month period	Repeated tester (Tested both beyond and within a 12-month period)
	% (95% CI)	% (95% CI)	% (95% CI)	% (95% CI)
All	45.2 (40.1–50.5)	33.8 (29.3–38.7)	13.7 (10.2–18.1)	7.2 (5.1–10.1)
Educational Level^a^				
Up to 9 years	38.4 (30.2–47.3)	14.8 (8.5–24.6)	42.7 (27.7–59.2)	16.8 (5.8–40.1)
Between 10 and 12 years	34.3 (27.5–41.8)	38.2 (30.8–46.3)	35.8 (23.1–50.8)	45.0 (28.6–62.6)
University level <3 years	16.8 (12.5–22.3)	25.7 (19.8–32.6)	10.8 (5.2–21.3)	25.6 (14.3–41.5)
University level ≥3 years	10.5 (7.5–14.6)	21.3 (16.3–27.2)	10.7 (5.6–19.4)	12.5 (6.0–24.1)
Country of birth [Sweden]	86.4 (77.6–92.1)	89.9 (81.6–94.7)	88.1 (74.7–94.9)	92.4 (74.1–98.1)
Having either parent born Sweden [Yes]^a^	75.0 (66.0–82.3)	81.5 (72.9–87.9)	59.3 (42.8–74.0)	82.7 (62.3–93.3)
Marital status [Married]	4.2 (2.4–7.3)	4.8 (2.5–9.2)	–	4.2 (0.6–24.5)
Municipality [Stockholm]^a^	33.0 (26.2–40.6)	48.0 (40.1–55.9)	39.4 (25.6–55.1)	61.7 (42.9–77.6)
Age (years), mean (SE)^b^	24.0 (23.6–24.5)	26.2 (25.7–26.6)	23.8 (22.8–24.7)	25.9 (24.9–26.9)
Self-perceived STI risk^a^				
No risk	61.3 (53.0–68.9)	67.9 (60.1–74.9)	30.8 (18.5–46.6)	27.3 (15.0–44.4)
Low risk	23.8 (17.6–31.4)	23.7 (17.8–30.8)	46.6 (31.7–62.1)	34.7 (20.1–52.9)
Medium or high risk	14.9 (9.45–22.6)	8.4 (4.6–14.8)	22.6 (13.0–36.3)	38.0 (22.6–56.3)
Social support				
Received help [Yes]^a^	89.5 (83.5–93.5)	98.4 (95.7–99.4)	97.5 (90.3–99.4)	95.8 (75.4–99.4)
Have someone share feelings [Yes]^a^	82.6 (75.6–87.9)	94.0 (89.6–96.6)	81.9 (68.0–90.5)	85.6 (67.9–94.3)
Trust in institutions				
Trust in healthcare [High]	75.7 (67.7–82.3)	80.4 (73.1–86.1)	65.3 (49.3–78.4)	87.8 (70.0–95.7)
Trust in school [High]	64.5 (56.5–71.8)	62.9 (54.7–70.5)	62.9 (47.6–76.1)	78.1 (61.9–88.6)
Trust in media [High]^a^	22.7 (16.6–30.3)	30.9 (23.8–39.0)	40.8 (26.5–56.9)	52.6 (35.4–69.3)

a Statistically significant (p < 0.05), Chi-squared test.

b Statistically significant (p < 0.05), F-test.

Regarding social capital, the percentage of young men who reported having received help from someone or having someone to share their inner feelings with was higher among those testing only beyond a 12-month period versus the other STI testing categories. The only institutional trust variable that significantly varied between STI testing categories was trust in media, which was lower for those never testing than for those belonging to the other STI testing categories (Table 2, p < 0.05).

### 4.3 Multivariable analyses

The weighted multivariable multinomial logistic regression models considered *“Never tested”* and negative answers to social capital questions as reference categories. This information is implicit in the results’ interpretation to avoid repetition.

**Fig. 1:**
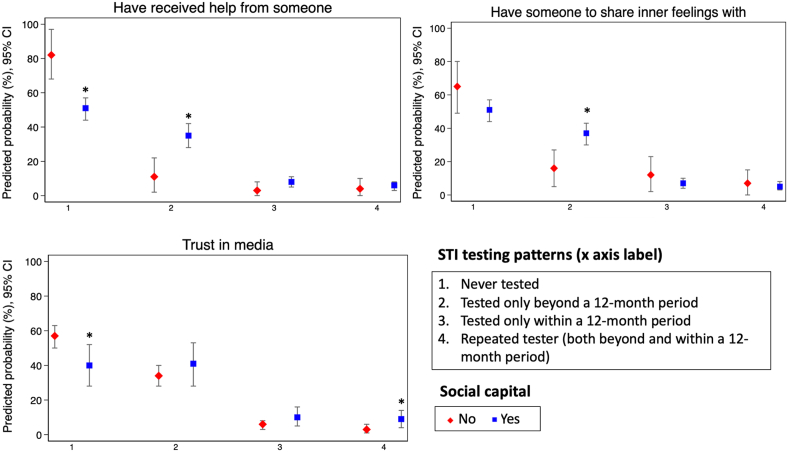
Association between different forms of cognitive social capital and STI testing patterns; predicted probabilities and 95% confidence intervals shown; Stockholm County, Sweden, 2017 (n = 467). *p < 0.05.

Table 3 shows that the multivariable analysis revealed that social support and trust in media increased the relative probability of being tested for STIs. Those who received help from someone (aRPR: 5.2, 95% CI: 1.7–16.2) or had someone to share inner feelings with (aRPR: 3.1, 95% CI: 1.2–7.7) had a higher relative probability of having been tested for STIs only beyond a 12-month period. Trust in media increased the relative probability of being tested only within a 12-month period (aRPR: 2.6, 95% CI: 1.1–6.1) and among repeated testers (aRPR: 3.6, 95% CI: 1.5–8.8) (Table 3, p < 0.05). The predicted probabilities of the significant social capital variables are shown in Fig. 1.

**Table 3:** Association between respondents’ social support, confounders, and sexually transmitted infections (STI) testing patterns; crude and adjusted weighted relative probability ratios (aRPR)‡ and 95% confidence intervals (CI) shown; Stockholm County, Sweden, 2017 (n=467)§.

Predictors	STI testing patterns					
	Tested only beyond a 12-month period		Tested only within a 12-month period		Repeated tester (Tested both beyond and within a 12-month period)	
	Crude weighted model	Adjusted weighted model	Crude weighted model	Adjusted weighted model	Crude weighted model	Adjusted weighted model
	RPR (95% CI)	aRPR (95% CI)	RPR (95% CI)	aRPR (95% CI)	RPR (95% CI)	aRPR (95% CI)
**Exposure**						
Received help [Yes]	7.1 (2.3–22.0)†	5.2 (1.7–16.2)†	4.6 (1.0–21.5)†	3.5 (0.6–19.4)	2.7 (0.3–21.2)	1.9 (0.3–12.7)
Have someone share feelings [Yes]	3.3 (1.6–6.9)†	3.1 (1.2–7.7)†	0.9 (0.4–2.2)	1.0 (0.3–2.8)	1.2 (0.4–3.8)	0.8 (0.2–3.0)
Trust in healthcare [High]	1.3 (0.7–2.3)	1.0 (0.5–1.9)	0.6 (0.3–1.3)	0.7 (0.3–1.8)	2.3 (0.7–7.6)	1.8 (0.5–6.6)
Trust in school [High]	0.9 (0.6–1.5)	0.7 (0.4–1.3)	0.9 (0.5–1.9)	0.8 (0.3–1.9)	1.9 (0.8–4.6)	1.5 (0.6–3.8)
Trust in media [High]	1.5 (0.9–2.6)	1.7 (0.9–3.3)	2.3 (1.1–5.0)†	2.6 (1.1–6.1)†	3.8 (1.7–8.4)†	3.6 (1.5–8.8)†
**Confounders**						
Educational Level						
Up to 9 years	REF	REF	REF	REF	REF	REF
Between 10 and 12 years	2.9 (1.3–6.3)†	2.3 (1.0–5.1)†	0.9 (0.4–2.2)	1.5 (0.5–4.7)	3.0 (0.8–11.2)	2.0 (0.5–7.6)
University level <3 years	3.9 (1.8–8.8)†	2.4 (1.0–5.8)†	0.6 (0.2–1.6)	0.7 (0.2–2.6)	3.5 (0.9–13.3)	1.6 (0.4–6.6)
University level ≥3 years	5.2 (2.3–11.7)†	2.0 (0.8–5.0)	0.9 (0.3–2.4)	1.2 (0.3–5.6)	2.7 (0.7–11.1)	0.8 (0.1–3.8)
Country of birth						
Other	REF	REF	REF	REF	REF	REF
Sweden	1.4 (0.6–3.5)	0.6 (0.2–2.2)	1.2 (0.4–3.5)	1.9 (0.4–10.0)	1.9 (0.4–9.2)	1.2 (0.1–12.4)
Having either parent born in Sweden						
No	REF	REF	REF	REF	REF	REF
Yes	1.5 (0.8–2.8)	1.5 (0.6–3.4)	0.5 (0.2–1.1)	0.3 (0.1–1.1)	1.6 (0.5–5.0)	1.2 (0.2–6.3)
Marital status						
Married	REF	REF	REF	REF	REF	REF
Unmarried	0.9 (0.4–2.1)	1.8 (0.6–5.4)	---	---	1.0 (1.2–8.0)†	0.8 (0.1–8.0)
Municipality						
Other (within Stockholm County)	REF	REF	REF	REF	REF	REF
Stockholm city	1.9 (1.2–3.0)†	1.3 (0.8–2.1)	1.3 (0.6–2.7)	1.5 (0.7–3.4)	3.3 (1.4–7.5)†	3.5 (1.3–9.4)†
Age (years), mean (SE)	1.3 (1.2–1.4)†	1.3 (1.1–1.4)†	1.0 (0.9–1.1)	1.0 (0.9–1.2)	1.3 (1.1–1.5)†	1.3 (1.1–1.6)†
Self-perceived STI risk						
No risk	REF	REF	REF	REF	REF	REF
Low risk	0.9 (0.5–1.5)	1.2 (0.6–2.1)	3.9 (1.7–8.9)†	4.2 (1.8–10.0)†	3.3 (1.3–8.5)†	4.6 (1.5–13.6)†
Medium or high risk	0.5 (0.2–1.2)	0.6 (0.2–1.6)	3.0 (1.2–7.8)†	2.6 (0.8–8.2)	5.7 (2.1–15.7)†	7.7 (2.5–24.3)†

‡ Model controlled for cognitive social capital questions, educational level, country of birth, either parent born in Sweden, marital status, municipality, age, and STI risk perception.

§ Reference category: Never tested.

† Statistically significant (p<0.05), Wald test.

The association between demographic characteristics and STI testing also varied by testing pattern. Compared to those reporting up to 9 years of education, all other educational levels increased the relative probability of testing but only among those testing only beyond a 12-month period. Those living in Stockholm municipality had higher relative probability of testing than those living in other municipalities within Stockholm County (aRPR: 3.5, 95% CI: 1.3–9.4). However, this association was only significant among repeated testers (Table 3, p < 0.05).

As age increased, the relative probability of testing also increased, and it was significant for testing patterns groups except those testing only within a 12-month period. Finally, compared to those reporting no self-perceived risk of acquiring an STI, those reporting low, medium, or high risk presented higher relative probability of testing but only among those testing only within a 12-month period and among repeated testers (Table 3, p < 0.05).

### 4.4 Population attributable fraction

The percentage of STI testing attributable to social support ranged from 63.7% (having someone to share inner feelings with) to 79.4% (having received help). Concerning trust in institutions, trusting in media was attributed to 25.1% of being tested for STIs only within a 12-month period and 38.1% of being tested for STIs both beyond and within a 12-month period.

## 5 Discussion

Our findings showed that different forms of social support increased the relative probability of being tested for STIs only for those who tested beyond a 12-month period. Trust in media increased the relative probability of two STI testing patterns (being tested only within a 12-month period and repeated testers). Although trust in healthcare and school was high (>50%), no statistically significant increase was found in the relative probability of STI testing.

### 5.1 Cognitive social capital by means of social support

Our study found that social support was a key factor facilitating STI testing, albeit only for those testing beyond a 12-month period. Our findings are similar to a previous qualitative study in this setting that reported that the association between social support and STI testing varied according to young men's discourses towards it (indifferent, ambiguous, or proactive) [49]. In Larsson et al.’s study, young men who had a proactive attitude towards testing had normalized this behavior and did not need peer support to test. However, for those with an ambiguous or indifferent stance, having trustworthy peers with whom they could share their concerns facilitated STI testing because trusted peers could provide emotional support, clarify doubts, provide health information, and encourage their friends to test when in need (social influence pathway) [49]. It is possible that in our sample, those testing beyond a 12-month period overlapped with those with an ambivalent or indifferent attitude towards testing. We also argue that the same pathways of change described in Larsson et al. [49] apply to our quantitative findings, as Larsson et al.'s data was collected in the same setting with participants within the same age range and sex.

Our findings are also consistent with studies conducted among MSM populations in the United States [50] and Canada [34] that report increased HIV testing among those with higher social capital. Such studies displayed how, for instance, higher levels of social capital not only predicted HIV [34,50] and other STI [34] testing among MSM, but also worked as a buffer to the stress generated by stigmatization within this group [34].

Through the PAF analyses, our study also found social support to be a major enabling factor to STI testing. Over 60 % of the STI testing among those testing beyond a 12-month period could be attributable to this form of social capital. Therefore, incorporating social support into interventions to promote STI testing in Sweden can result in positive outcomes given the important prevalence of this factor in the study population and its positive relationship with being tested.

### 5.2 Cognitive social capital by means of trust in institutions

In our study, trust in institutions was hypothesized to be a key enabling factor for STI testing. However, even though participants in our study reported an overall high trust in healthcare and school, and low trust in media, only trust in media facilitated STI testing. The lack of a statistically significant relationship between trust in healthcare and STI testing found in our paper diverges from studies conducted elsewhere showing that mistrust in healthcare [51,52] was negatively associated with health service utilization.

One explanation for this finding could be the low variability of the variable measuring trust in healthcare in our study. The low variability might reflect the overall high trust in the healthcare system that has been reported before in this setting [53,54]. In addition, our trust variable neither inquired about which specific healthcare aspects were trusted (professionals, the system, interventions, etc.) nor the reasons behind it, which could have provided detailed information for use in the analyses. Further studies are needed to explore more in-depth if trust in healthcare institutions enables STI testing among young men in Stockholm.

Trust in school did not statistically increase the relative probability of being tested for STIs in our study. Although sexual education (including STI prevention) is compulsory in Sweden, its quality varies greatly across schools [55]. It is also unclear to what extent information about STI services is consistently discussed across the country. This might explain why the high trust in school does not translate into higher testing rates. Nevertheless, given the good level of trust in school reported by our participants and the possibility of reaching teenagers before their sexual initiation, schools might be a key setting for sexual health interventions promoting STI testing.

Trust in media was the only statistically significant institutional trust factor facilitating STI testing. Although our study did not measure what type of media was trusted (online, printed, radio, etc.), our findings are in line with a scoping review reporting that most interventions using different forms of social media improved sexual health outcomes, including testing for STIs among young people [56]. Thus, considering that 25%–38% of STI testing in our study was attributed to trust in media, interventions developed to facilitate young men's STI testing in this setting should include different media outlets. Further quantitative and qualitative studies are needed to explore in-depth the pathways by which trusting in media increases STI testing, as well as which specific media format (online, printed, radio, etc.) can explain this association.

Our data also showed that as age increased, the relative probability of STI testing increased. One possible explanation of this finding is that as age increases, young men are more likely to have more sexual partners, increasing their STI risk and thus their need for testing [57]. Living in Stockholm municipality also increased the relative probability of testing when compared to those living in other municipalities in Stockholm County, but only among those testing repeatedly. This finding might signal differences in geographical access to STI testing services among young men. Further studies are needed to map the magnitude and reasons behind this finding.

Self-perceived risk of STI testing also increased the probability of testing but only among those testing within a 12-month period or those testing repeatedly. The relationship between self-perceived risk and STI testing has also been reported in a recent systematic review [58]. The fact that the aforementioned association was significant only among those testing recently or repeatedly can be explained by the temporality of the measurement used in this study. We inquired about current self-perceived risk, which is likely to be more important for current testing patterns than for older ones.

Our analysis showed that higher formal educational levels increased the relative probability of STI testing only among those testing beyond a 12-month period. This is a surprising finding since we expected formal education to be a predictor of testing for all testing patterns. One possible explanation is that the effect of formal education on STI testing is mediated by other demographic characteristics not measured in this study. For example, one study conducted among females in the United States found that the association between formal education and STI testing varied depending on the race of the sample [59]. Thus, more studies are needed to understand why the association between formal educational levels and STI testing varies according to the testing patterns found in our study.

### 5.3 Public health implications

As Bhattacharaya described, “multiple levels of social relationship interact, often overlapping in their influences on both safety-enhancing and risk-promoting sexual behaviors” [60]. Hence, improving individuals’ social capital is not the only step to improving sexual health in a population through spontaneous cooperation. It is crucial to also ensure that these individuals are receiving and sharing trustworthy and non-stigmatized information with their peers, thus shaping peer group social norms to improve the dissemination of healthy sexual behaviors. The high level of trust in healthcare and school, as well as the increase in the relative probability of being tested among those who trust in media, as presented in this study, highlights the potential to use such institutions to encourage young men to undergo STI testing in Sweden.

Interventions aiming to use individual social capital to improve STI testing should combine different forms of it. This is because media, healthcare, and educational institutions (vertical information sources) often provide accurate information, but only friends, family, and acquaintances (horizontal information sources) can offer information that is tailored to a person's specific needs, which has been shown to be crucial for information effectiveness [61].

### 5.4 Strengths and limitations

To the best of our knowledge, this study is the first one to assess the association between cognitive social capital (social support and institutional trust) and STI testing in Sweden. In addition, our analysis allowed us to identify STI testing patterns that have not been reported before. This is important since it allowed us to capture a more detailed description of STI testing behaviors among young males.

However, our study also has important limitations. First, the cross-sectional nature of our data does not allow us to establish a temporal association between the exposures and outcome. Second, the generalization of our findings is limited due to the low response rate and the underrepresentation in our final sample of young men with low educational level, immigrant background, and those not living in Stockholm city. Although we used probability weights to correct for the above, residual bias can be present since other unknown characteristics that differentiate respondents and non-respondents were not measured in our study [62]. Third, it is known that small sample sizes decrease the power of the analyses, which was reflected in larger confidence intervals [63],pp20]. Fourth, although this study used demographic information obtained from reliable and accurate sources [64], the number of "married" individuals in our sample might have been underestimated. This is because it is common for individuals living in Sweden to share the same household in a marriage-like form without being officially married [65]. Therefore, some people who are living together may not be registered as "Married" in official records.

Finally, it is possible that the prevalence of STI testing in our study is overestimated since respondents were more educated, native, and older individuals than non-respondents. These characteristics have been shown to increase testing in a previous study [10]. On the other hand, the effect of social capital on STI testing might be underestimated for those born abroad, as high social capital could compensate for cultural and language differences influencing access to healthcare services.

## 6 Conclusions and recommendations

Our study showed that young men in Stockholm County have different STI testing patterns. Social support and trust in media were factors enabling STI testing; however, this association varied according to the young men's STI testing pattern. Trust in media increased the relative probability of testing among those testing recently (within a 12-month period) and those testing repeatedly, whereas social support increased testing only among those testing beyond a 12-month period. Our findings highlight the importance of social capital in facilitating STI testing among young men living in Stockholm County. Further studies are needed to identify and explore in-depth the mechanisms by which trust in media increases STI testing in this population. Due to the risk of selection bias and limited generalizability, this study's findings should be interpreted with caution.

## Ethics statement

Ethical approval was obtained from the Stockholm Regional Ethics Committee (reference number 2017/833-31/5). Participation was voluntary, and the survey included the essential elements of informed consent.

## Data availability

The authors have followed the European Union General Data Protection Regulation (2016/679) regarding restricted access to the data. The sensitive nature of this study's data means that even if the names and other identifying information (address, telephone number, etc.) of the young men part of this research have been removed from the dataset, there is a possibility that they could be identified thorough other variables. Requests for access to the data should be made to the Research Data Office at Karolinska Institutet via rdo@ki.se, and if permitted by law and ethical approval, decided on a case-by-case basis, the data can be shared.

## Author contribution statement

Ana Paula Finatto Canabarro: Conceived and designed the experiments; Performed the experiments; Analyzed and interpreted the data; Wrote the paper. Zangin Zeebari: Performed the experiments; Analyzed and interpreted the data; Wrote the paper. Malin Eriksson; Anna Nielsen: Analyzed and interpreted the data; Wrote the paper. Mariano Salazar: Conceived and designed the experiments; Performed the experiments; Analyzed and interpreted the data; Contributed reagents, materials, analysis tools or data; Wrote the paper.

## Declaration of competing interest

The authors declare the following financial interests/personal relationships which may be considered as potential competing interests: Mariano Salazar reports financial support was provided by Forskningsrådet för hälsa arbetsliv och välfärd (FORTE).

Acknowledgments

## Figures and Tables

**figs1:**