# Supplementary material for: Cognitive social capital as a health-enabling factor for STI testing among young men in Stockholm, Sweden: A cross-sectional population-based study
Source: Heliyon. 2023 Oct 8;9(10):e20812. doi: 10.1016/j.heliyon.2023.e20812 (PMC10590937; doi:10.1016/j.heliyon.2023.e20812)
Supplement: Multimedia component 1 [file mmc1.docx]

**Supplementary material**

**Appendix 1.** Translated version of the questions used for this survey.

| **Testing and risk perception questions** | | | | |
| --- | --- | --- | --- | --- |
| 1. Have you tested yourself, or bought a test, for a sexually transmitted infection (ex.: chlamydia, gonorrhea, syphilis, HIV, trichomonas or other) in some of the following places? | | | | |
| 1. Youth health clinic | Never 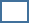  Yes, during the last 12 months 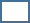  Yes, over 12 months ago 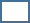 | | | |
| 1. Primary healthcare center | Never 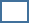  Yes, during the last 12 months 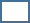  Yes, over 12 months ago 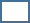 | | | |
| 1. Private healthcare clinic | Never 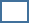  Yes, during the last 12 months 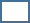  Yes, over 12 months ago 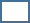 | | | |
| 1. Hospital/Infectious disease clinic/Venereal disease clinic | Never 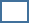  Yes, during the last 12 months 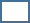  Yes, over 12 months ago 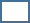 | | | |
| 1. Non-cost chlamydia test available online | Never 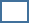  Yes, during the last 12 months 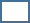  Yes, over 12 months ago 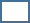 | | | |
| 1. Chlamydia test available at the pharmacy. | Never 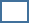  Yes, during the last 12 months 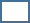  Yes, over 12 months ago 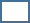 | | | |
| 1. Other | Never 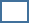  Yes, during the last 12 months 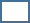  Yes, over 12 months ago 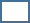 | | | |
| 2. Have you tested, or bought a test, specifically for chlamydia? | Never 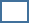  Yes, during the last 12 months 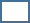  Yes, over 12 months ago 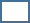 | | | |
| 3. How do you perceive your current risk of becoming infected with chlamydia or other sexually transmitted infections? | No risk  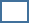 | Low risk  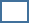 | Medium risk  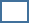 | High risk  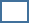 |

| **Social capital questions** | |
| --- | --- |
| 4. In the last 12 months, have a friend or acquaintance helped or performed any service to you? | Yes 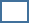 No 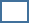 |
| 5. Do you have someone you can trust and share your innermost feelings with? | Yes 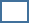 No 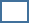 |
| 6. What confidence do you have in the following institutions in society? | |
| 1. Healthcare | Very little 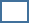 Quite little 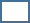  Quite a lot 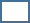 A lot 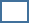 |
| 1. School | Very little 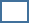 Quite little 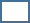  Quite a lot 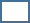 A lot 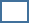 |
| 1. Media | Very little 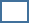 Quite little 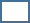  Quite a lot 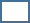 A lot 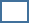 |
